# A potent betulinic acid analogue ascertains an antagonistic mechanism between autophagy and proteasomal degradation pathway in HT-29 cells

**DOI:** 10.1186/s12885-016-2055-1

**Published:** 2016-01-16

**Authors:** Debasmita Dutta, Biswajit Chakraborty, Ankita Sarkar, Chinmay Chowdhury, Padma Das

**Affiliations:** Cancer Biology and Inflammatory Disorder Division, CSIR-Indian Institute of Chemical Biology, 4 Raja S. C. Mullick Road, Kolkata, 700032 India; Organic and Medicinal Chemistry Division, CSIR-Indian Institute of Chemical Biology, 4 Raja S. C. Mullick Road, Kolkata, 700032 India

**Keywords:** Apoptosis, Autophagy, Betulinic acid analogue, Proteasomal pathway

## Abstract

**Background:**

Betulinic acid (BA), a member of pentacyclic triterpenes has shown important biological activities like anti-bacterial, anti-malarial, anti-inflammatory and most interestingly anticancer property. To overcome its poor aqueous solubility and low bioavailability, structural modifications of its functional groups are made to generate novel lead(s) having better efficacy and less toxicity than the parent compound. BA analogue, 2c was found most potent inhibitor of colon cancer cell line, HT-29 cells with IC_50_ value 14.9 μM which is significantly lower than standard drug 5-fluorouracil as well as parent compound, Betulinic acid. We have studied another mode of PCD, autophagy which is one of the important constituent of cellular catabolic system as well as we also studied proteasomal degradation pathway to investigate whole catabolic pathway after exploration of 2c on HT-29 cells.

**Methods:**

Mechanism of autophagic cell death was studied using fluorescent dye like acridine orange (AO) and monodansylcadaverin (MDC) staining by using fluorescence microscopy. Various autophagic protein expression levels were determined by Western Blotting, qRT-PCR and Immunostaining. Confocal Laser Scanning Microscopy (CLSM) was used to study the colocalization of various autophagic proteins. These were accompanied by formation of autophagic vacuoles as revealed by FACS and transmission electron microscopy (TEM). Proteasomal degradation pathway was studied by proteasome-Glo™ assay systems using luminometer.

**Results:**

The formation of autophagic vacuoles in HT-29 cells after 2c treatment was determined by fluorescence staining – confirming the occurrence of autophagy. In addition, 2c was found to alter expression levels of different autophagic proteins like Beclin-1, Atg 5, Atg 7, Atg 5-Atg 12, LC3B and autophagic adapter protein, p62. Furthermore we found the formation of autophagolysosome by colocalization of LAMP-1 with LC3B, LC3B with Lysosome, p62 with lysosome. Finally, as proteasomal degradation pathway downregulated after 2c treatment colocalization of ubiquitin with lysosome and LC3B with p62 was studied to confirm that protein degradation in autophagy induced HT-29 cells follows autolysosomal pathway.

**Conclusions:**

In summary, betulinic acid analogue, 2c was able to induce autophagy in HT-29 cells and as proteasomal degradation pathway downregulated after 2c treatment so protein degradation in autophagy induced HT-29 cells follows autolysosomal pathway.

## Background

Natural products serve an important role and are used as starting point in drug discovery program. Thus, nature has been a source of medicinal agents for thousands of years and an impressive number of modern drugs have been isolated from natural sources [1]. In fact, a majority of anticancer and anti-infectious agents are of natural origin [2, 3].

Despite the obvious benefits of chemo treatment, which is an effective drug treatment designed to kill cancer cells in individuals, there are several adverse side effects to this form of treatment that should be considered in every cancer treatment strategy as they tend to have various therapeutic effects and patients may ultimately die due to multiple organ failure. Therefore development of alternative potent therapeutic agents having minimal side effects is of current interest [4].

Today, numerous natural compounds extracted from plants source are reported to possess growth inhibitory effects on various tumor cells. Many medicinal plants have been found as potential sources of many pharmaceuticals possessing diversified biological activities [5] and most of these bioactive compounds have negligible toxicity. Thus, plants are the reservoirs of a large number of important organic compounds and they have long been used traditionally as the sources of medicines to cure or prevent diseases [6]. The medicinal properties of plants could be defined based on the antioxidant, antimicrobial, antipyretic effects and others effects of the phytochemicals present in them [7]. As compared to synthetic compounds, natural compounds have more structural diversity and novelty and many natural chemicals are able to interact with proteins, and other biological molecules. Also, it is more complex in structure than synthetic molecules. This complexity allows for more selective binding to targets.

One such natural compound is Betulinic acid (3β-hydroxy-lup-20(29)-en-28-oic acid), methanolic extract of *Dillenia indica* fruits, a lupane class type, naturally occurring pentacyclic triterpenoid. It has antiretroviral, anti-malarial and anti-inflammatory properties, as well as a more recently discovered potential as an anticancer agent, by inhibition of topoisomerase [7].

Earlier report suggest that one characteristic feature of betulinic acid’s cytotoxicity is its ability to trigger the mitochondrial pathway of apoptosis which causes cancer cell death [8]. It is reported that betulinic acid induces apoptosis in tumor cells which is accompanied by caspase activation, mitochondrial membrane alterations and DNA fragmentation [9]. Similarly, we had earlier reported that betulinic acid analogue, 2c induced apoptosis is accompanied by ROS generatlion, phosphatidyl serine exposure to outer membrane, chromatin condensation and DNA fragmentation [10].

In the present endeavour, we targeted to study another classical form of PCD, autophagy as drug-induced autophagy is progressively reported as a cause to induce cell death. At the same time we also considered that autophagy is one of the important pathways for cell death processes. Two major pathways accomplish regulated protein catabolism in eukaryotic cells: the autophagy-lysosomal system which involves the sequestration of plasmatic portions and intracellular organelles into double-membrane vacuoles called autophagosomes and the ubiquitin-proteasome system, the primary route of degradation for thousands of short-lived proteins play a crucial role in monitoring other basic cellular processes, like normal protein turnover, protein quality control by degrading misfolded and damaged proteins, metabolism, cell death, cell cycle control etc. [11]. Ubiquitin, a small globular protein containing 76 amino acid residues is covalently attached as a degradation signal to other proteins which are going to be degraded in an ATP-dependent manner and these ubiquitinated proteins are generally delivered to proteasomes. Recognition of ubiquitinylated proteins is mediated by p62/SQSMT1, the first protein reported to have such an adaptor function. Besides, p62 possesses a C-terminal ubiquitin-binding domain (UBA) [12] by which it interacts with ubiquitin noncovalently and a short LIR (LC3-interacting region) sequence responsible for LC3 interaction [13]. It is known that p62 is required for the clearance of ubiquitinylated proteins and it may also deliver ubiquitinylated cargos to the proteasome besides autolysosomes but they are mainly degraded by autophagy [14, 15] and thus plays essential roles for their autophagic clearance [16, 17]. Activation of proteasomal degradation pathway is usually inversely correlated with autophagic degradation.

Generally, activation of autophagy refers to cellular survival strategy whereas its persistent activation may lead to cell death [18]. In this study, we demonstrate some promising results obtained from a betulinic acid analogue, 2c in HT-29 colon carcinoma cells. Interestingly, it induced autophagy by activating Atg proteins, LC3 conversion and autophagosome formation.

**Scheme 1:**
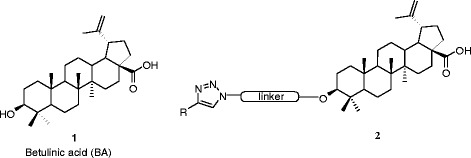
Betulinic acid (**1**) and its designed analogue, **2c** (**2**)

Our study shows that the analogue 2c has potent anticancer activity in relation to HT-29 cell line (Scheme 1).

## Methods

### Antibodies and reagents

Pen strep, RPMI 1640, DMEM, Heat inactivated Fetal Bovine Serum (FBS), Lyso Tracker® Red DND-99 were purchased from Invitrogen (Carlsbad, CA, USA). The antibodies against β-Actin, Alkaline phosphatase/ Horseradish peroxidase conjugated secondary antibodies and enhanced chemiluminescence kit were purchased from Santa Cruz Biotechnology (Santa Cruz, CA, USA). The antibodies against Beclin-1, LC3, Atg 3, Atg 5, Atg 7, Atg5-Atg 12, p62, LAMP-1, Ubiquitin were purchased from Cell Signaling Technology (Inc. Beverly, MA, USA). Rapamycin was procured from Enzo Life Sciences (Farmingdale, NY) as part of the Cyto-ID® Autophagy Detection Kit. Alexa Fluor-633 and Alexa Fluor-488 were obtained from Life Technologies (Carlsbad, CA, USA). Z-Val-Ala-DL-Asp (methoxy)-fluoromethylketone (Z-VAD-FMK) was obtained from BD Biosciences (San Jose, CA, USA). All other chemicals were obtained from Sigma-Aldrich (St. Louis, Missouri, USA).

### Cell lines

HT-29-colon carcinoma (an adherent cancer cell line) and HCT-15-Human colon adenocarcinoma (an adherent cancer cell line) were obtained from National Centre for Cell Sciences, Pune, India and maintained in RPMI-1640 medium. The media were supplemented with 10 % FBS and antibiotics (50 IU/ml penicillin G and 50 μg/ml streptomycin). The cells were incubated at 37 °C in a humidified incubator containing 5 % CO_2_ and subcultured every 72 h using an inoculum of 5 × 10^5^ cells/ml. Cell viability (>95 %) was confirmed by trypan blue exclusion.

### Materials

3-(4,5-Dimethylthiazol-2-yl)-2, 5-diphenyl-tetrazolium bromide (MTT) was purchased from USB Corporation (USA). Pen strep, RPMI 1640, High Glucose DMEM, and Heat inactivated Fetal Bovine Serum (FBS), 5,5′,6,6′-tetrachloro-1,1′,3,3′- tetraethyl benzimidazolyl carbocyanine iodide (JC-1), and 5-(and-6)-chloromethyl-2′,7′-dichloro dihydrofluorescein diacetate (CM-H_2_DCFDA) were obtained from Invitrogen (Carlsbad, CA, USA). Caspase-3, Caspase-8, Caspase-9 colorimetric assay kits were procured from Biovision (Milpitas, CA, USA). The antibodies against Bcl2, Bcl-xl, Bax, Bad, β-Actin, and PARP, Alkaline phosphatase/Horseradish peroxidase conjugated secondary antibodies, and enhanced chemiluminescence kit were purchased from Santa Cruz Biotechnology (Santa Cruz, CA, USA).

### Cell viability assay

The cytotoxic activity of 2c dissolved in DMSO (final DMSO concentration <0.1 %) was assessed in HCT-15 using MTT assay. At first, cells (1.25–2.5 × 10^4^ cells/100 μl of RPMI 1640 or high glucose DMEM medium/well) were cultured in 96-well tissue culture plates followed by treatment with betulinic acid or its derivatives dissolved in DMSO (using 0–50 μM concentration) for 48 h at 37 °C, 5 % CO_2_. Thereafter, cell viability was measured by adding 20 μl MTT (5 mg/ml in PBS) and incubated for 4 h at 37 °C. Subsequently, 100 μl DMSO was added to each well, resultant optical densities were measured at 540 nm in an ELISA Reader (BIO RAD, CA, USA). The specific absorbance that represented formazan production was calculated by subtraction of background absorbance from total absorbance. The mean percentage viability was calculated as follows:

$$ \frac{\mathrm{Mean}\ \mathrm{specific}\ \mathrm{absorbance}\ \mathrm{of}\ \mathrm{treated}\ \mathrm{cells}}{\mathrm{Mean}\ \mathrm{specific}\ \mathrm{absorbance}\ \mathrm{of}\ \mathrm{untreated}\ \mathrm{cells}}\times 100 $$

The results were expressed as IC_50_ values which were enumerated by graphical extrapolation using Graph Pad Prism software (version 5, Graph Pad Prism software Inc, San Diego, CA, USA). Each experiment was performed at least three times and in duplicate.

### Autophagy flux measurement

The method is based on Cyto-ID staining of autophagic compartments (pre-autophagosomes, autophagosomes, and autophagolysosomes) in live cells using Cyto-ID® Autophagy Detection Kit. Autophagic compartments are determined as intermediate constituents of a dynamic lysosomal degradation process and their intracellular abundance at a particular time point is a function of the established equilibrium between their generation and degradation. Autophagic flux established the discrimination between early induction of autophagosome formation and late inhibition of autophagosome maturation results in an ultimate increase in autophagosomal presence. Autophagy was measured by staining autolysosomes and autophagic compartments with the fluorescent probe Cyto-ID® Green (Enzo Life Sciences, Farmingdale, NY) as recommended by manufacturer. In Cyto-ID assay the specific dye selectively stains autophagic compartments and therefore allows determination of autophagic flux as accumulation of stained compartments. Samples were then analyzed in the green (FL1) channel of the FACS Caliber flow cytometer. Briefly, HT-29 cells (10^5^ to 10^6^cells/ml) were treated with analogue 2c (IC_50_; 14.9 μM) and positive control rapamycin (1–5 μmol/L; 24 h) followed by washed with PBS. Cyto-ID Green containing indicator was added to the cell culture free medium, containing 5 % FBS. Cyto-ID Green concentration contains 1 μl of Cyto-ID Green Detection Reagent in 1 ml cell culture medium. It was then mixed well and incubated for 30 min under standard tissue culture conditions at 37 °C, 5 % CO_2_ in the dark. At the end of staining procedure, the Cyto-ID containing medium was washed with PBS. Then trypsinization was done and after washing cells were resuspended in ice cold PBS and staining was performed. Autophagy was measured by percent autophagosome formation [19].

### Acidic vesicular organelles detection

A basic evidence of autophagy induced cells is gradual formation of Acidic vesicular organelles (AVO) [20]. Acridine orange, a weak base that traverses freely across biological membranes was used to stain AVOs in autophagic cells. When there is no appearance of AVO, AO remains in an uncharged state which shows green fluorescence. 2c treated (IC_50;_ 14.9 μM; 0–48 h) and control HT-29 cells (2.5 × 10^5^/ml) were washed in PBS and incubated with AO (1 μg/ml) for 15 min at 25 °C [21]. Cells were again washed with PBS. AVO formation was observed using fluorescence microscope at an excitation of 488 nm and emission of 530 and 650 nm.

### Monodansylcadaverine (MDC) staining

Autophagic vacuoles were detected with Monodansylcadaverine (MDC), a fluorescent compound which is incorporated in multilamellar bodies by two ways i.e. ion trapping mechanism and interaction with membrane lipids, used as a probe for detection of autophagic vacuoles (which are part of the lysosomal compartment) in cultured cells.

Briefly, after treatment with analogue 2c (IC_50;_ 14.9 μM) HT-29 cells were treated with PBS and then incubated with 0.05 mM of MDC (prepared in hot methanol) at room temperature for 1 h. After incubation, the cells were again washed three times with PBS and immediately analyzed by fluorescence microscopy (Olympus IX70) under 40× magnification using an excitation filter of 360 nm and an emission filter of 525 nm [22].

### Effect of 3-MA on 2c induced cytotoxicity

To study the effect of autophagy inhibitor 3-MA in 2c induced cytotoxicity, HT-29 cells (2.5 × 10^4^/100 μl of RPMI 1640 medium / well) were pre-incubated with 3-MA (10 mM) for 4 h before the addition of IC_50_ concentration of 2c for 48 h at 37 °C, 5 % CO_2_. Thereafter, 20 μl MTT (5 mg/ml in PBS) was added and subjected to measure cell viability after incubation for 4 h at 37 °C. Subsequently, 100 μl DMSO was added to each well, resultant optical densities were measured at 540 nm in an ELISA Reader (BIO RAD, CA, USA). The specific absorbance that represented formazan production was calculated by subtraction of background absorbance from total absorbance. The mean percentage viability was calculated as follows:

$$ \frac{\mathrm{Mean}\ \mathrm{specific}\ \mathrm{absorbance}\ \mathrm{of}\ \mathrm{treated}\ \mathrm{cells}}{\mathrm{Mean}\ \mathrm{specific}\ \mathrm{absorbance}\ \mathrm{of}\ \mathrm{untreated}\ \mathrm{cells}}\times 100 $$

The results were determined as IC_50_ values which were enumerated by graphical extrapolation using Graph Pad Prism software (version 5, Graph Pad Prism software Inc, San Diego, CA, USA). Each experiment was performed at least three times and in duplicate.

### Western blotting analysis

Control and 2c treated (IC_50_; 14.9 μM; 0–48 h) cells were lysed in lysis buffer (50 mM Tris–HCl, pH 7.4, 150 mM NaCl, 1 mM EDTA, 1 mM EGTA, 1 μg/ml protease inhibitor cocktail, 5 mM PMSF and 1 mM DTT containing 1 % Triton X-100), sonicated and centrifuged for 10 min at 4 °C at 10,000 × g and protein concentration estimated. Electrophoretic separations (50 μg protein/ lane) were carried out on 10 % SDS-polyacrylamide gel electrophoresis and electrotransferred onto a PVDF membrane. Blots were blocked for 1 h at 37 °C in 20 mM Tris-HCl, pH 7.4, 150 mM NaCl, 0.02 % Tween 20 (TBST) containing 5 % skimmed milk and probed using 1:2000 dilution of appropriate antibodies (β-Actin, Beclin-1, Atg 3, Atg 5, Atg 7, Atg 5–12, p62) by incubating overnight at 4 °C. The membranes were washed thrice with TBST, incubated with alkaline phosphatise / Horseradish peroxidase conjugated secondary antibody and the bands visualized using a 5-bromo-4-chloro-3-indolyl phosphate / nitro blue tetrazolium substrate or enhanced chemiluminescence kit. For further quantification of protein bands their Densitometric analysis was performed using the software Image J as required. To study the effects of various autophagic inhibitors, whole cell lysates were prepared from control and 2c treated [(14.9 μM; 0, 48 h) Bafilomycin A1 (50 nM), E64d (10 μg/ml) with pepstatin A (10 μg/ml), Chloroquine (5 μM)] HT-29 cells, protein concentration estimated and western blotting analysis was done as described above.

### Quantitative real-time PCR

Total RNAs, from the HT-29 cell line treated with analogue 2c and respective control were isolated using the Trizol method, purified and treated with DNase I. Briefly, 1 μg of total RNA from each sample was reverse transcribed using the random hexamar primer in a 20 μl reaction mixture. Each RNA sample was mixed with 400 ng of oligo-(dT)-P3 primer and incubated at 70 °C for 10 min. The mix (10 μl) was quickly chilled on ice and then mixed with equal volume of a mixture of 2× reverse transcriptase buffer, 8 mM dNTPs (with dTT), 20 U RNase inhibitor and 50 U MultiScribe™ reverse transcriptase (High capacity cDNA Reverse Transcription kit, Applied Biosystems) and reverse transcribed at 42 °C for 60 min followed by inactivation at 70 °C for 10 min. The mRNA expression was determined by quantitative PCR (qRT-PCR) on ABI 7000 PCR platform. For this assay, 100 ng of cDNA was used in a 10 μl reaction mixture with SYBR® Green PCR Master Mix (Applied Biosystems) and 25 ng of both forward and reverse primers. Conditions for quantitative PCR was 94 °C for 5 min, 40 cycles of 94 °C for 30 s, 55 °C for 30 s, and 72 °C for 30 s [23]. All samples were amplified in duplicate, and every experiment was repeated independently at least two times. Relative gene expression was determined using the 2−ΔΔCT method, with GAPDH as the internal control.

The following primers were used:

**Table:** 

Serial	Primer Name	Sequence (5′—3′)
1	GAPDH F HM	CTCTGCTCCTCCTGTTCGAC
2	GAPDHR HM	GTTAAAAGCAGCCCTGGTGA
3	Beclin1 F HM	TAGACCAGCTGGACACTC
4	Beclin1 R HM	CTTGCGGTTCTTTTCCAC
5	LC3B F M	ACGGCGCTTGCAGCTCAATG
6	LC3B R M	CGAGGCATAAACCATGTAC

### Transmission electron microscopy

Detecting the presence of autophagic vesicles by using transmission electron microscopy (TEM) is the most sensitive and gold standard technique to monitor autophagy. Control and analogue 2c treated (14.9 μM; 0–48 h) HT-29 cells (2.5 × 10^5^/ml) were fixed in 2.5 % glutaraldehyde and 2 % paraformaldehyde in 0.1 M phosphate buffer (pH 7.4) for 1 h at 4 °C. After rinsing in PBS, cells were post fixed in osmium tetroxide (1 %) for 2 h, dehydrated in graded acetone and embedded in araldite CY212. Semi thin sections were cut, stained with 0.5 % toluidine blue (5 min) and examined under a light microscope (Olympus, 60 ×). Ultrathin sections were stained with 2 % uranyl acetate and Reynold’s lead citrate, and observed with a transmission electron microscope (Technai G2) [24].

### Proteasomal degradation assay

The Proteasome-Glo™ Cell-Based Assay are homogeneous, luminescent assays that individually measure the chymotrypsin-like, trypsin-like or caspase like protease activity associated with the proteasome complex in cultured cells. The 26S proteasome is a 2.5 MDa multiprotein complex found both in the nucleus and cytosol of all eukaryotic cells and is comprised of a single 20S core particle and 19S regulatory particles at one or both ends [25]. Three major protealytic activities as chymotrypsin-like, trypsin-like and post-glutamyl peptide hydrolytic or caspase-like were determined by proteasome-Glo™ assay systems (Promega). Together these three activities are responsible for much of the protein degradation required to maintain cellular homeostasis including degradation of critical cell-cycle proteins, tumor suppressors, transcription factors, inhibitory proteins and damaged cellular proteins.

In brief, after treatment with 2c for 12, 24 and 48 h HT-29 cells were removed from T-75 cm^2^ flask using minimal (0.5–1.0 ml) amount of trypsin to flask surface and incubated just until cells detached. Then complete medium (10 % FBS) was added to cell suspension. After two additional washing with complete medium, 10,000 cells per well were plated in 96-well plate.

Proteasome-Glo™ Cell-Based Reagent was prepared before beginning the assay according to Manufacturer’s instruction. 100 μl of Proteasome-Glo™ Cell-Based Reagent was added to each 100 μl of sample and appropriate controls as needed. Then the plate was covered using a plate sealer. The contents of the wells were mixed at 700 rpm for 2 mins using a plate shaker and incubated at room temperature for a minimum period of 10 min. The luminescence of each sample was measured in a plate-reading luminometer. Proteasomal activities were normalized by total protein concentration.

### Laser scanning confocal microscopy

The autophagy regulated proteins namely LC3B, Beclin I, Atg 5, Atg 7, and adaptor protein P62 in the cytosol of autophagic cells and their co localization in autophagic pathway were analysed using confocal microscopy [26]. In brief, HT-29 cells were grown on cover slips. After treatment, cells were washed thrice with PBS. Then cells were fixed with 4 % paraformaldehyde for 15 min followed by permeabilized with 0.4 % Triton X-100 for 15 min at room temperature. After blocking with BSA for 1 h, cells were incubated overnight with primary antibodies of Beclin 1, Atg 5, Atg 7, LC3B, p62, Ubiquitin, LAMP-1 diluted in DPBS with 1 % BSA and 0.1 % Tween 20. Then cells were washed thrice with PBS and incubated with fluorescent tagged secondary antibodies atleast for 2 h. Alexa Fluor-633 and Alexa Fluor-488 fluorescent conjugated secondary antibodies were used. LysoTracker Red DND-99 was used to stain lysosomes in HT-29 cells. After rinsing in PBS 3 times, cells were finally counterstained with 1 mg/ml of 4,6-diamidino-2-phenylindole (DAPI) to visualize the nucleus for 5 mins and again washed with PBS for three times. Fluorescence signals were captured using laser scanning confocal microscope (Leica TCS SP2 System Leica Microsystem, Heidelberg, Germany, using 100×). At least 20 randomly selected microscopic fields were observed per sample.

### Statistical analysis

The statistically significant differences between control and drug-treated cells were calculated using one way ANOVA. Multiple comparisons were made between different treatments (analysis of variance) using Graph Pad Prism Software (version 5, GraphPad Software Inc, San Diego, CA, USA). All the experiments were carried out in triplicate and values were reported as mean ± SD. Student’s t test was used for determining statistical significance (*P* <0.05). Software Origin 8.5, Image J were used for preparation of different bar diagrammatic representations and measurement of intensities of images, blots respectively.

## Results

### Cytotoxic activity of Betulinic Acid analogue, 2c on HCT-15

The cytotoxic activity of betulinic acid analogue, 2c was studied using MTT assay on HCT-15. We assessed the effects of different concentrations (0–50 μM) of 2c for 48 h. As in our previous study, **2c** deciphering highest cytotoxicity to HT-29 cells, anticancer activity of **2c** was also measured against another Human colon adenocarcinoma, HCT-15 cell line and interestingly IC_50_ value was found 21.6 ± 1.3 μM. Finally, as 2c deciphering lowest IC_50_ against HT-29, its role as an inducer of autophagy was investigated only in HT-29 cell line.

### Autophagy flux detection: % autophagosome formation

**Fig. 1:**
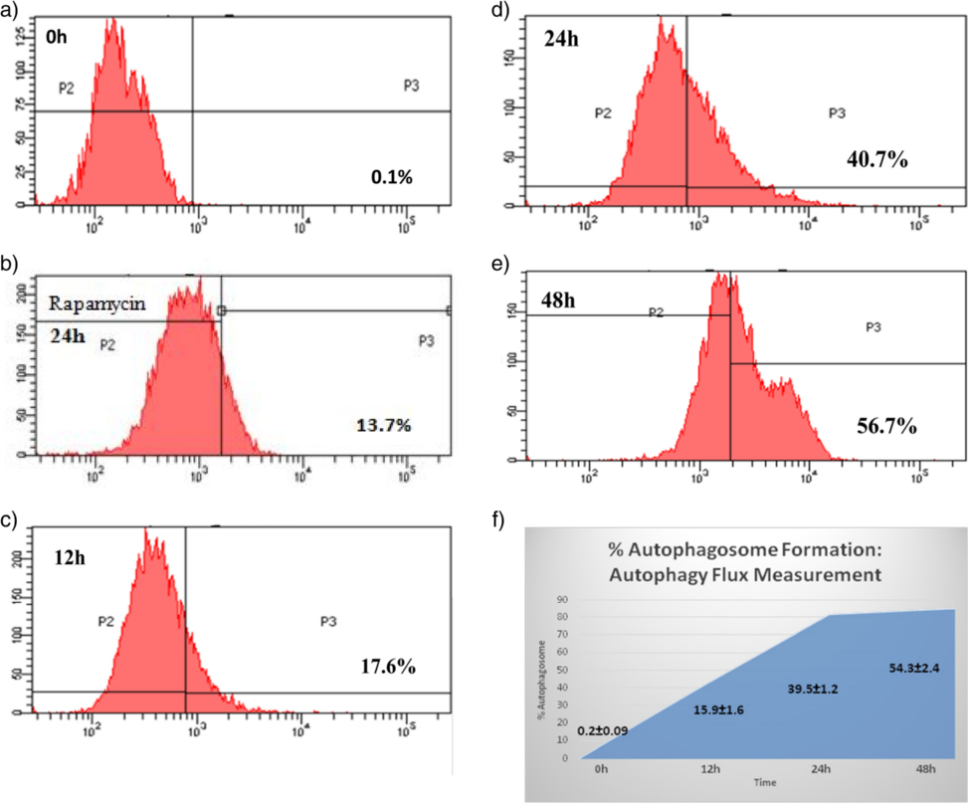
Autophagy Flux Measurement. HT-29 cells were treated with 2c for different time intervals (12, 24, 48 h) and one set with Rapamycin (positive control) for 24 h only. Autophagy was measured by staining autophagosomes and autophagic compartments with the fluorescent probe Cyto-ID® Green. **a** Control cells (**b**) positive Control and 2c treated HT-29 cells of (**c**) 12 h, (**d**) 24 h, (**e**) 48 h were stained and analyzed in the green (FL1) channel of the FACS Caliber flow cytometer. **f** The graphical representation of autophagosome formation shown increasing percentage of autophagosomes with respect to control cells according to the different time periods. This indicates increase in autophagy flux formation in a time dependent manner. The figures are representative profile of at least three experiments

Authophagy induction can be examined by another established method where a Cyto-ID Green dye was selectively used to label autophagosomes and then the presence of autophagosome in HT-29 cells was analysed by flow cytometry. Autophagosomes were stained with the Cyto-ID autophagy detection kit as described in materials and method. Cyto-ID Green dye was used to selectively label autophagosome and the percentage of Cyto-ID-positive cells correlates with the number of autophagosome so we measured the percentage of Cyto-ID-positive cells by flow cytometry with respect to different time period of incubation with the lead analogue, 2c. As shown in Fig. 1, the Flow cytometry analysis clearly reveals that 2c treatment in HT-29 cells increased the number of autophagosomes in a dose dependant manner (12, 24 and 48 h) as compared to control indicating 2c induces autophagy in HT-29 cells. The cells were also treated with rapamycin (positive control) for 24 h and we found an increased percentage of Cyto-ID-positive cells as compared to control suggesting that rapamycin, an established inducer of autophagy also causes increased percentage of Cyto-ID-positive cells.

### 2c induces AVO formation

**Fig. 2:**
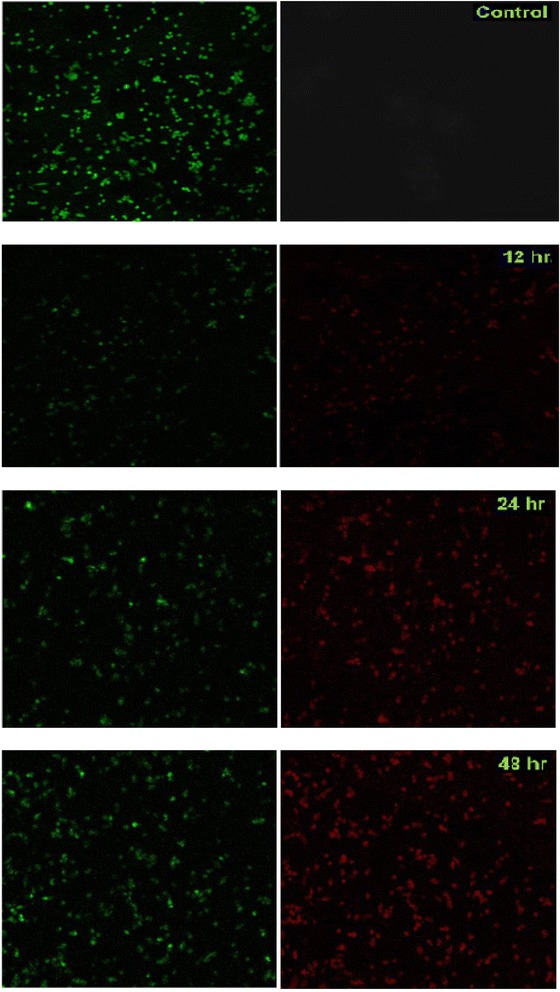
Formation of AVO. Control and 2c (14.9 μM; 0–48 h) treated HT-29 cells (2.5 × 10^5^/ml) were stained with acridine orange (1 μg/ml) for 15 min and AVO formation was measured using fluorescence microscope (60×)

Acidic vesicular organelles (AVO) formation js a well established feature of autophagic cells [27]. Acridine Orange (AO) is a lysotropic dye that accumulates in acidic organelles in a pH-dependent manner. At neutral pH, Acridine Orange is a hydrophobic green fluorescent molecule. Within acidic vesicles, Acridine Orange becomes protonated and trapped within the organelles. Protonated Acridine Orange forms aggregates that emits bright red fluorescence. We visualized the effect of betulinic acid analogue 2c on AVO generation in HT-29 cells after its staining with the lysosomotropic agent Acridine orange by fluorescence microscopy as described in materials and methods. As control cells do not contain any AVO, they only displayed green fluorescence without any red fluoresorescence. When HT-29 cells were treated with 2c for different time periods gradual increase of red fluorescence was observed in a time dependent manner. Our data reveals maximum red fluorescence observed after 48 h of treatment indicating maximum number of AVO formation (Fig. 2).

### Labeling of autophagic vacuoles with monodansylcadaverine (MDC)

**Fig. 3:**
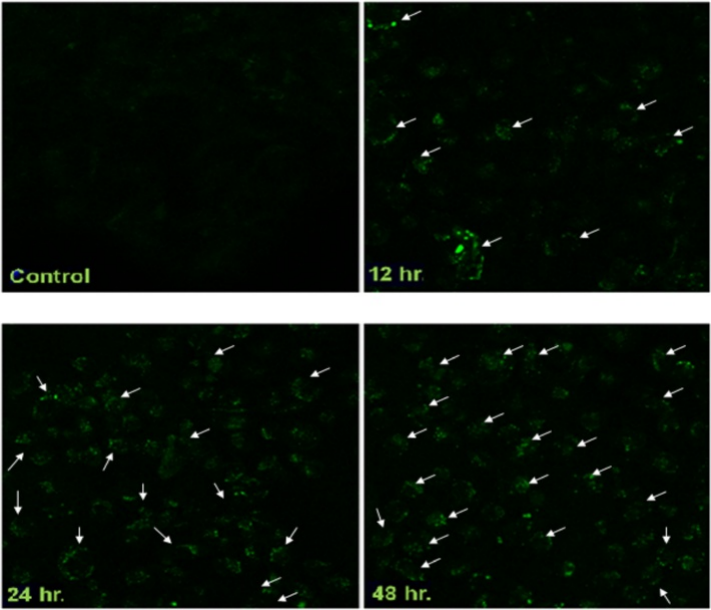
2c induced vacuolization and formation of MDC-labeled vesicles in HT-29 cells. Cells were incubated in RPMI 1640 medium. After 2c treatment with indicated time intervals, both treated and control cells (0 h) were incubated with MDC at 0.05 mM for 10 min at 37 °C followed by washing with PBS (four times) and immediately analyzed under fluorescence microscopy where the nature of the vacuoles was confirmed to be authophagic (40× magnification) with increasing intensity with respect to different time periods

We next assessed whether analogue 2c induced autophagy in HT-29 cells. Earlier reports suggests that MDC accumulate in mature autophagic vacuoles such as autophagolysomes but not in the early endosomes compartments [28]. Furthermore, autophagic vacuoles stained by MDC appear as distinct dot like structure which is distributed in the cytoplasm. MDC accumulation in autophagic vacuoles is due to a combination of ion trapping and specific interactions with vacuole membrane lipids. We studied the incorporation of MDC stain in 2c treated HT-29 cells for different duration of time (12, 24 and 48 h) by fluorescence microscopy. As shown in Fig. 3 MDC labeled vacuoles were scarcely detected in control cells, whereas the cells which were treated with 2c, clearly showed numerous MDC labeled fluorescent vacuoles with an increasing intensity with respect to different time intervals indicating that 2c treatment in HT-29 cells induced the formation of the MDC labeled autophagic vacuoles.

### 2c causes alteration in autophagic proteins level

**Fig. 4:**
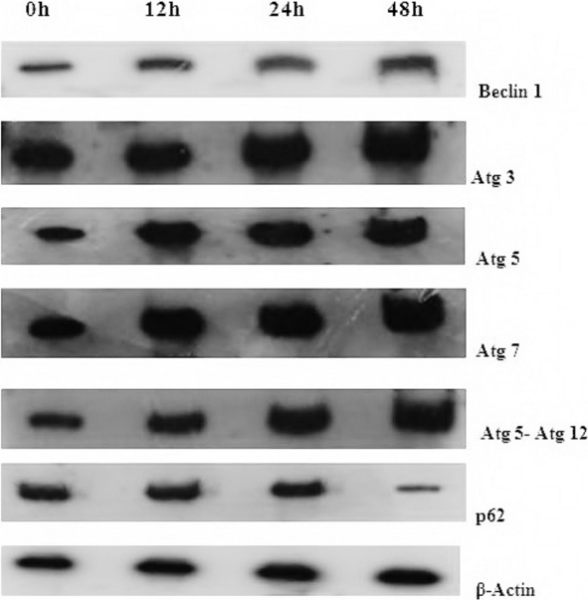
Expression of Autophagy proteins in 2c induced HT-29 cells. Cells were treated with 2c (14.9 μM for 12, 24, 48 h) and expression levels of Beclin-1, Atg 3, Atg 5, Atg 7, Atg 5-Atg 12, p62 were quantified by western blot analysis from cell lysates of control and treated cells. Analysis was confirmed with three different sets of experiments. β-actin served as a loading control

Atg proteins are fundamental proteins engaged in autophagic pathway from initiation to maturation step and play crucial role in autophagosome formation [29]. Beclin 1, the mammalian orthologue of yeast Atg 6, is a key regulatory protein in autophagic pathway. Bcl-2 family proteins interact with Beclin1, inhibiting it through binding with its BH3 domain and ultimately causes inhibition of autophagy. Therefore, up regulation of Beclin1 family of protein with Atg proteins is another indicator of autophagy. We studied the expression level of different autophagic proteins by western blotting as described in materials and methods. The expression levels of the Beclin 1, Atg 5, Atg 3, Atg 7, Atg 5-Atg 12 proteins were increased and p62 level was decreased with analogue 2c treatment (Fig. 4). Beclin 1 is required for initiating the formation of autophagic vacuoles [30].

### 2c induces conversion of LC3

**Fig. 5:**
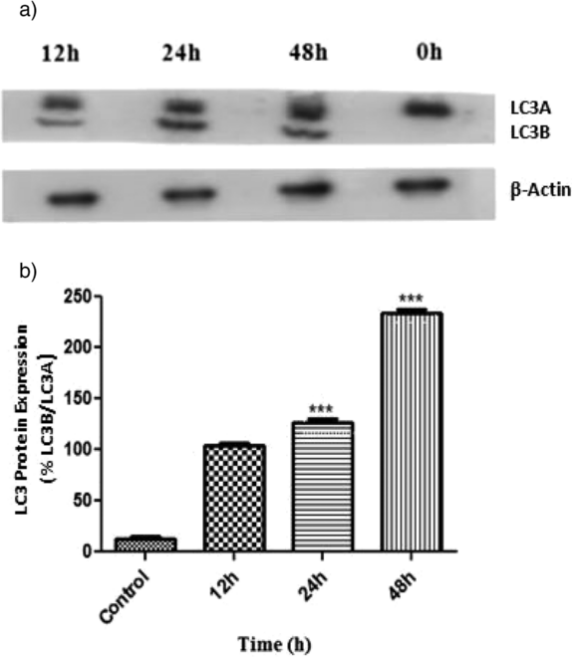
Conversion of LC3A to LC3B and densitometric analysis. **a** Control and 2c treated (14.9 μM; 0–48 h) whole cell lysates of HT-29 cells were prepared and analysed by western blotting to study the conversion of LC3A to LC3B. β-actin was used as loading control. The figure is a representative profile of at least three experiments. **b** Bands obtained from western blotting were quantified by densitometric analysis and expression level of LC3 protein was determined. Histograms represent LC3 protein expression normalized to β-actin (***p <0.001)

Recent investigations suggest that there are two forms of LC3 protein LC3A and LC3B [31]. LC3A is cytoplasmic form and is processed into LC3B which is autophagsome membrane bound. Hence the amount of LC3B is correlated with the extent of autophagosome formation. In our study after treatment with analogue 2c for 12, 24 and 48 h in HT-29 cells, the expression level of LC3A (18 kDa) and LC3B (16 kDa) was investigated. Western blot displayed gradual appearance of LC3B after 12, 24 and 48 h treatment with respect to control (Fig. 5a). All protein expression levels confirm occurrence of autophagy. We further confirm this data with densitometric analysis which demonstrated that level of LC3B/ LC3A protein relative to β-Actin increased significantly after 24 and 48 h of 2c treatment (***p <0.001) with respect to control (Fig. 5b).

### Effects of various autophagic inhibitors on 2c induced autophagy in HT-29 Cells

**Fig. 6:**
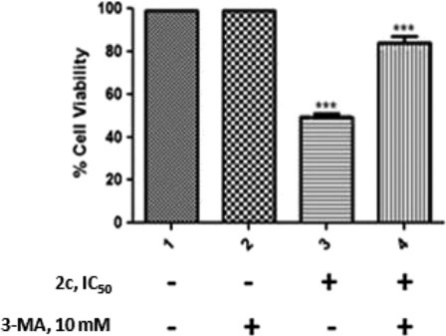
Effect of autophagy inhibitor 3-MA in 2c induced cytotoxicity. HT-29 cells (2.5 × 10^4^/100 μl of RPMI 1640 medium / well) were pre-treated with autophagy inhibitor, 3-MA (10 mM) for 4 h followed by 2c treatment for 48 h. Cell viability was measured by MTT assay. Histograms represent percentage cell viability (Mean ± SEM) obtained at the IC_50_ concentration of 2c (14.9 μM) and has been derived from at least three experiments in duplicate (***p <0.001 compared to only 2c treated cells)

**Fig. 7:**
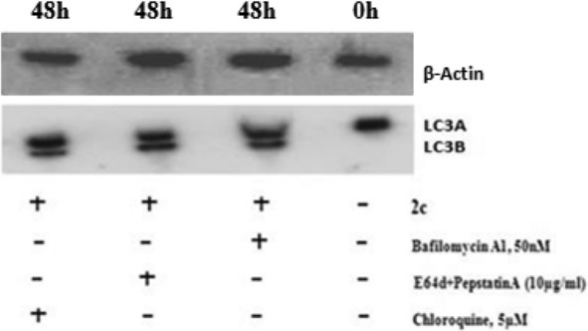
Effect of Bafilomycin A1, Chloroquine, E64d+PepstatinA. HT-29 cells were pre-incubated with Bafilomycin A1(50 nM), Chloroquine (5 μM) and E64d+PepstatinA (10 μg/ml) for 4 h and then treated with 2c (14.9 μM; 48 h). After preparation of whole cell lysates, western blotting was done using specific antibodies against LC3B. Analysis was confirmed with three different sets of experiments

3-methyladenine (3-MA) interferes with autophagy initiation by blocking Class III PI3K, an activator of autophagy which plays a crucial role in the early step of autophagosome formation i.e. responsible for autophagosome biogenesis in mammalian cells [32]. So, we were interested to find out the role and contribution of 2c in autophagy induced cell death. HT-29 cells were pretreated with 3-MA (10 mM; 4 h), which blocks autophagy initiation and then incubated with IC_50_ concentration of 2c for 48 h. 3-MA significantly attenuated 2c induced cytotoxicity in HT-29 cells (Fig. 6). Bafilomycin A1, chloroquine and pepstatin A+E64d are used to block autophagic progression by impairing lysosomes. Monitoring LC3B conversion by Western blot analysis in the presence of different lysosomal degradation inhibitors, such as bafilomycin A1, chloroquine and pepstatin A+E64d [33], is a hallmark experiment to detect progression of autophagic flux. It is reported that when autophagic flux is induced, the level of LC3B is increased in the presence of a lysosomal degradation inhibitor as the degradation of LC3B through autolysosomal compartment will no longer possible [34]. The conversion of LC3B significantly increased in presence of each lysosomal degradation inhibitor after 48 h of treatment with 2c (Fig. 7).

### 2c causes alteration in mRNA expression level of key autophagic proteins

**Fig. 8:**
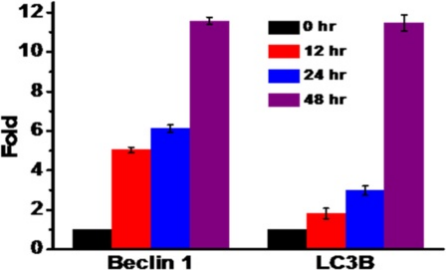
2c-induced expression of Beclin1 and LC3B mRNA. 2c treated and untreated HT-29 cells were subjected to RNA isolation and the expression of mRNA was analysed. Beclin1 and LC3B expression in HT-29 cells treated with or without 2c were measured by qRT-PCR and normalized to GAPDH expression. Data are expressed as fold change relative to untreated cells showing induction of autophagy in cells treated with 2c for different time intervals (12, 24 and 48 h)

Upregulation of mRNA expression of specific autophagic proteins, induce autophagy. In this experiment, RNAs were isolated from 2c treated HT-29 cells and the mRNA expression levels of Beclin1 and LC3B were measured by quantitative real-time PCR as described in materials and methods. The cells were treated with analogue 2c (IC_50_; 14.9 μM) for different time intervals (0–48 h) then Beclin1 and LC3B expression relative to GAPDH were determined by real-time PCR [35]. As shown in Fig. 8, incubation with 2c increased the relative expression of Beclin1 and LC3B mRNA in HT-29 cell lines according to the various time periods.

### Quantification of autophagic vacuoles using TEM

**Fig. 9:**
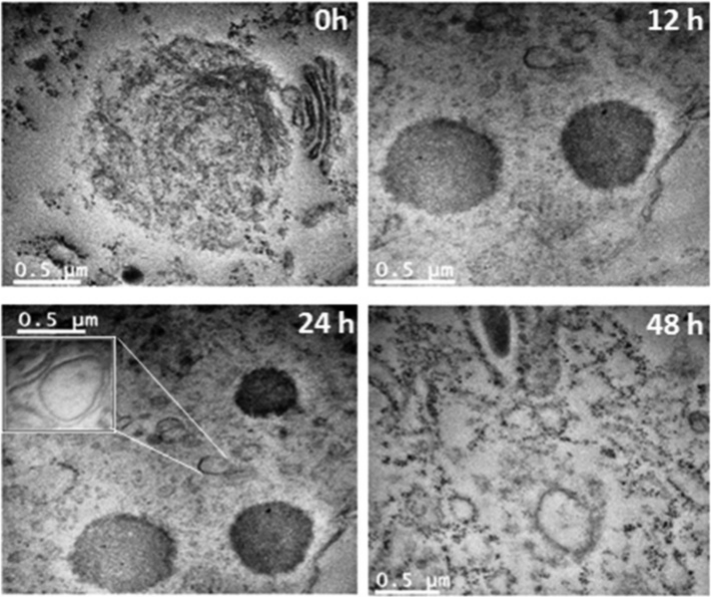
TEM microphotographs of 2c treated HT-29 cells. Ultra structure of control and 2c (14.9 μM; 12, 24, 48 h) treated HT-29 cells shows formation of double membrane autophagic vacuoles. The figure is a representative profile of at least three experiments

Quantification of double-membrane vacuoles in autophagic cells using TEM is a gold standard method to confirm occurance of autophagy. We have already showed by light microscope that 2c treatment in HT-29 cells (48 h) causes formation of cytoplasmic vacuoles and we further confirm this result using transmission electron microscopy, which demonstrated that control cells do not contain any vacuoles while ultrastructure of 2c treated (12, 24 and 48 h) cells showed presence of large vacuoles (Fig. 9). These double membraned vacuoles ultimately fused with lysosomes resulting in the formation of autolysosomes.

### 2c causes declination of proteasome degradation pathway

**Fig. 10:**
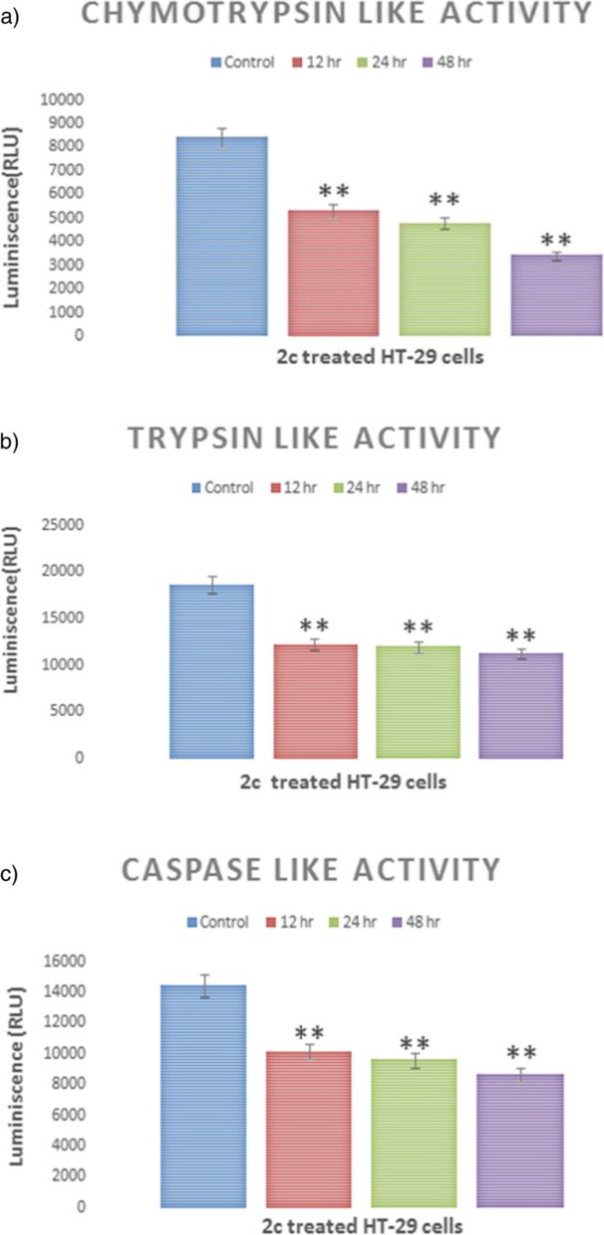
2c treatment causes declination of Proteasome degradation pathway. HT-29 cells were treated with 2c and then subjected to measure three subtypes of proteasomal activities. As shown in figure there is a gradual time dependent decrease in the proteasomal activities i.e. downregulation of (**a**) chymotrypsin-like, (**b**) trypsin-like and (**c**) caspase-like enzyme activities occurs with respect to control according to different time intervals (12, 24, 48 h). Values are mean ± S.D and represent at least three experiments (**P <0.05 compared to control)

Extensive evidence has shown that there is a connection between the two protein degradation pathway namely ubiquitin proteasome system (UPS) and autophagy. Autophagy complements the UPS for the degradation of polyubiquinated proteins [36]. Evidences suggest that activation of proteasomal degradation pathway is inversely proportional to the activation of autophagic pathway. Inhibition of the proteasome causes induction of autophagy. The proteasome has three distinct ATPase-independent protealytic activities, namely, caspase-like, trypsin-like and chymotrypsin-like activities, which can be attributed to the β1, β2 and β5 subunits respectively, within the constitutive proteasome of the 20S core barrel-like structure of the proteasome that has two outer heptameric rings of α subunits and two inner heptameric rings of β subunits in mammalian cells. In this experiment, the caspase-like, trypsin like and chymotrypsin-like activities of the proteasome were assayed by a chemiluminescence-based method. The induction of autophagy by 2c treatment reduced all three subtypes of proteasomal protealytic activities in HT-29 cells (Fig. 10). From our data, it is clearly visible that downregulation of trypsin-like, chymotrypsin-like and caspase-like occurs with respect to control at various time intervals (12, 24 and 48 h).

### Immunostaining of different autophagic proteins and their colocalization

Western Blot analysis of autophagic proteins prompted us for further investigation to finally confirm all the hallmark phenomenon of autophagic pathway. Autophagy is mainly monitored by Atg family of proteins and LC3B expression considered as a convincing marker of autophagy can be detected by confocal microscopy.

**Fig. 11:**
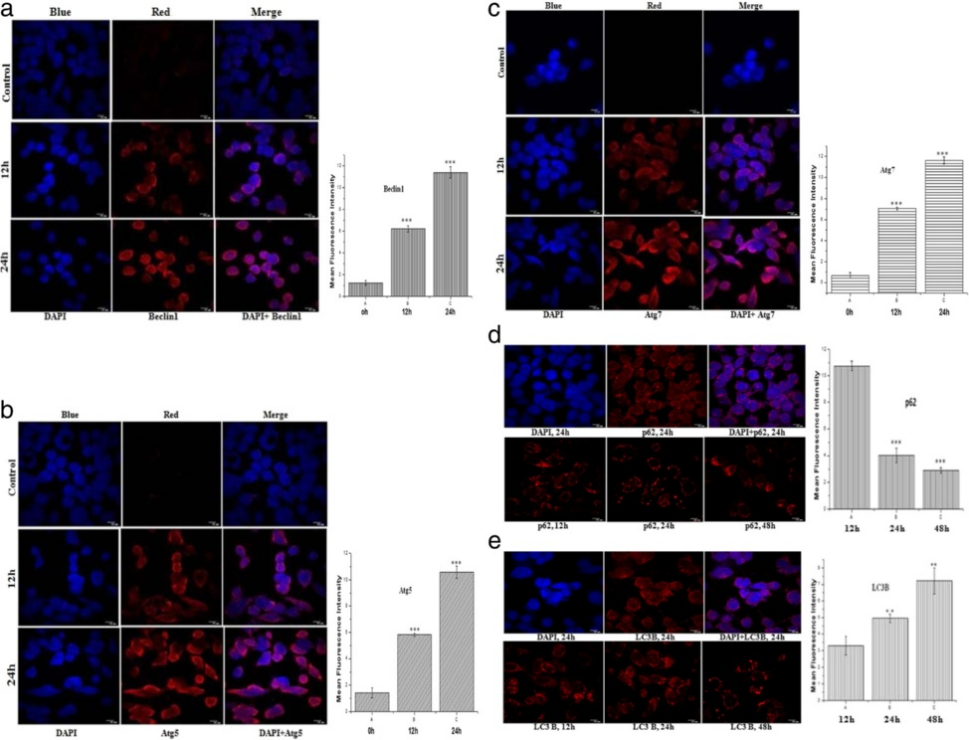
Timecourse analysis of different autophagic proteins expression with immunostaining. Representative Confocal Laser Scanning Microscopy (CLSM) images showing HT-29 cells were treated with 2c for autophagic component like Beclin-1(12 and 24 h), Atg-5(12 and 24 h), Atg-7(12 and 24 h), p62(12, 24, 48 h) and LC3B(12, 24, 48 h) fixed and stained with respective fluorescent tagged secondary antibody of (**a**)Beclin1 (*red*), (**b**) Atg5 (*red*), (**c**) Atg7 (*red*), (**d**) p62 (*red*) (**e**) LC3B (*red*) and counter stained with DAPI (*blue*). Graphs depict quantification of intensity of Beclin1, Atg5, Atg7, LC3B and p62 expression level respectively in HT-29 cells at indicated time points after 2c incubation. Values are expressed as Mean Fluorescence Intensity ± SEM of three independent experiments (**p <0.01, ***p <0.001). Scale bars, 10 mm

To substantiate whether Beclin1, the central protein of autophagic pathway expressed in HT-29 cells after 2c treatment for 12 and 24 h with respect to control we labeled Beclin1 with red and nucleus with blue fluorescence (DAPI). Immunofluorescence in HT-29 cells revealed that gradual increase of expression level of autophagy regulated protein in compared with corresponding control (Fig. 11a). Furthermore, we similarly analysed the expression levels of Atg5, Atg7 (Fig. 11b, c) and found enhanced level of protein expression with progression of time. We next sought to determine p62, an adaptor protein was labeled with red fluorescence and nucleus was counterstained with DAPI for 24 h. The adaptor protein p62 recognizes ubiquitinylated proteins during autophagy by its ubiquitin receptors interacting with ubiquitin noncovalently via their ubiquitin-binding domains. This p62 can deliver ubiquitinylated cargos to autophagolysosome for their degradation and is required for the aggregation of ubiquitinylated proteins. Furthermore, p62 itself is gradually eliminated with autophagic clearance [37]. As p62 plays essential roles in cellular ubiquitinylated proteins clearance and levels of p62 usually inversely correlate with autophagic degradation, we monitored p62 expression level for 12, 24 and 48 h (Fig. 11d) as confirming evidence towards progression of autophagy. As shown in Fig. 11d, p62 expression level was found to have gradually decreased with progression of 2c treatment time in HT-29 cells.

**Fig. 12:**
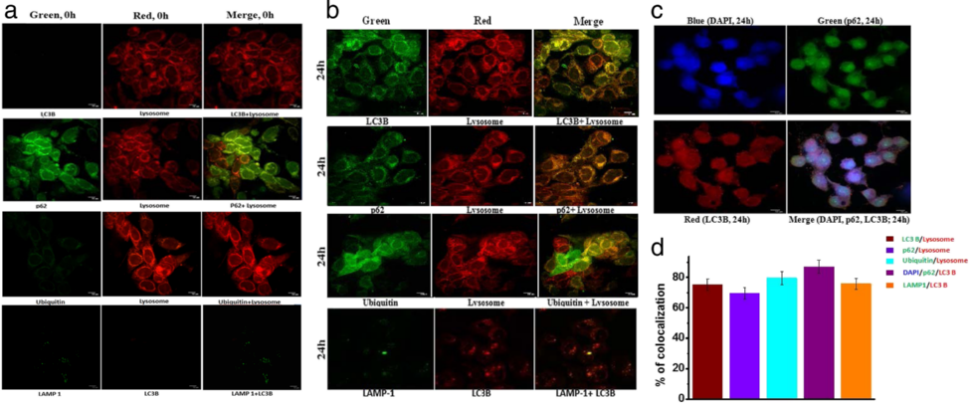
Co-localization of proteins associated with autophagy. Immunofluorescence (IF) demonstrates (**a**) colocalization of LC3B (*green*) with Lysosome (*red*), p62 (*green*) with Lysosome (*red*), Ubiquitin (*green*) with Lysosome (*red*), LAMP-1(*green*) with LC3B (*red*) and p62 (*green*) with LC3B (*red*) in HT-29 cells without **2c** treatment. **b** colocalization of LC3B (*green*) with Lysosome (*red*), p62 (*green*) with Lysosome (*red*), Ubiquitin (*green*) with Lysosome (*red*), LAMP-1(*green*) with LC3B (*red*) and (**c**) p62 (*green*) with LC3B (*red*) counterstained with DAPI (*blue*) in HT-29 cells treated with **2c** for 24 h. **d** percentage of cellular puncta co-localized for five pair of protein sets were determined. Scale bars, 10 mm. The bars represent Mean ± SEM

Conjointly all the stages of autophagy from formation of autophagosome to its fusion with lysosomes can be monitored with expression level of LC3B protein at different time point. Upon activation of autophagy, pro-LC3 is cleaved at its C-terminus to form LC3A and the free C-terminal glycine is then modified by lipidation to a faster migrating form, LC3B which is tightly associated with the autophagosomal membrane via conjugation to phosphatidylethanolamine [38]. Expression level of LC3B is directly correlate with progression of autophagy. We next performed immunofluorescence analysis for LC3B, expression level of which was found to increase in a time dependent manner (Fig. 11e). Furthermore, we studied the influence of analogue 2c on autophagy component which is playing key role in autophagy pathway like LAMP-1 (green)-LC3B (red); LC3B (green)-Lysosome (Lysotracker, red) and p62 (green)-LC3B (red)-DAPI (blue) by colocalization Finally as we have already shown proteasomal degradation pathway downregulated after 2c treatment so autolysosomal pathway may be the only system available for ubiquitinylated proteins degradation. The adaptor molecule, p62 delivers the ubiquitinylated cargos to autolysosome for degradation. So, colocalization of Ubiquitin (green)-Lysosome (Lysotracker, red) and p62 (green)-lysosome (Lysotracker, red) were studied to confirm whether protein degradation in autophagy induced HT-29 cells followed autolysosomal pathway (Fig. 12a, b, c).

As we know LC3B protein present on autophagosomes which ultimately fuses with lysosome so we stained LC3B with green fluorescence and lysosome with lysotracker red and found 75.2 % of their colocalization after 24 h of **2c** treatment whereas no LC3B expression was observed in case of control HT-29 cells (Fig. 12a, b, d). We next colocalize p62 (green) with Lysosome (Lysotracker, red) as p62 cargos polyubiquitinated proteins to autolysosome for degradation. p62 was colocalized with lysosome by 69.6 % after 24 h of **2c** treatment but no significant colocalization was noticed in untreated cells (Fig. 12a, b, d). Furthermore we interested to manifest this phenomenon by colocalization of ubiquitin (green) with lysosome (Lysotracker, red) as proteins which are destined for degrdation in autolysosome by p62 are all tagged with ubiquitin chain and in control cells ubiquitin expression was low but in **2c** treated HT-29 cells 79.8 % of their colocalization of ubiquitin with lysosome was observed (Fig. 12a, b, d). Finally, we again focused on p62 which possesses a C-terminal ubiquitin-binding domain (UBA) [39] and a short LIR (LC3-interacting region) sequence responsible for LC3 interaction. p62 serves as a linker in between LC3B on autolysosome and ubiquinated substrate. So, we were interested to check percentage of colocalization of p62 (green) with LC3B (red) as well as counterstained nucleus with DAPI (blue) and found 87.1 % of colocalization in 2c treated HT-29 cells (12b, c). LC3B expression was found negligible in control cells as autophagy was not induced in on the contrary p62 was significantly expressed (Fig. 12a). Lysosomal-associated membrane protein-1 (LAMP-1), the main constituent of lysosomal membrane, was measured to ensure autophagolysosome formation and assessed colocalization of LAMP-1 (green) with LC3B (red). No LC3B expression was found in control cells whereas 75.9 % of colocalization was obtained in 2c treated HT-29 cells after 24 h of treatment (Fig. 12a, b, d).

So, our findings suggest that protein degradation in autophagy induced HT-29 cells follows autolysosomal pathway and all polyubiquitinated proteins are reached to autolysosome by p62 for their degradation.

### 2c Causes independent occurrence of apoptosis and autophagy in HT-29 cells

**Fig. 13:**
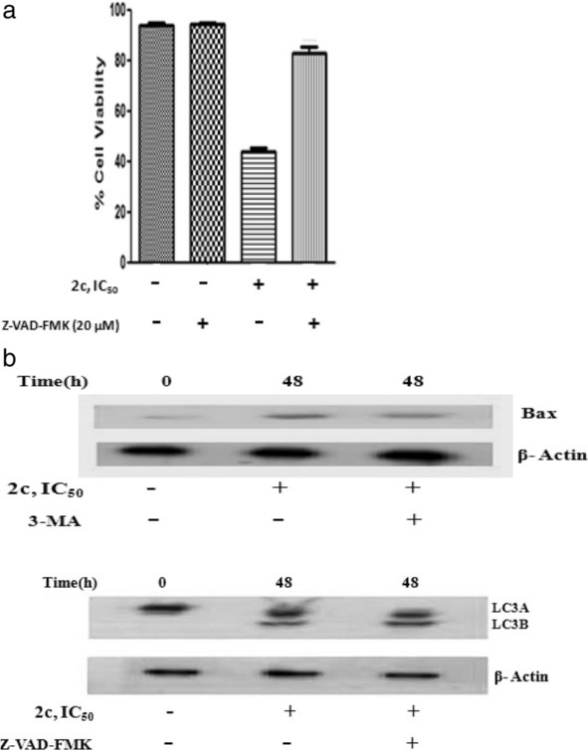
Effect of 3-MA and Z-VAD-FMK on apoptosis and autophagy. HT-29 cells were incubated with IC_50_ concentration of **2c** (48 h) after treatment with 3-MA (10 mM; 4 h) or Z-VAD-FMK (20 mM; 4 h) as well as untreated cells also. (**a**) Cell viability was measured after Z-VAD-FMK (20 mM, 4 h) treatment. (**b**) Whole cell lysates were prepared and subjected to immunoblot analysis using specific antibodies against Bax or LC3. Analysis was confirmed with three different sets of experiments

Previously, **2c** was found to induce apoptosis and all findings of this present study revealed **2c** as an inducer of autophagy in HT-29 cells. Consequently, crosstalking in these two signaling cascade was studied. The interplay between autophagy and apoptosis is complicated and differs with cell types and specific stress causing factors of cell death [40]. They may act independently to each other. To determine whether **2c** induced apoptosis and autophagy in HT-29 cells are independent or interconnected, we evaluated cells treated with **2c** in the presence of either apoptosis or autophagy inhibitors (Z-VAD-FMK and 3-MA respectively). After treatment with 3-MA (10 mM) for 4 h, cells were incubated with **2c** (14.9 μM; 48 h) followed by western blot analysis. Figure 13 revealed that in presence of autophagy inhibitor and **2c** treatment, Bax, a well known proapoptotic protein level remained same as comparable to that of cells treated only with **2c**. Similarly, cell lysates were pre-incubated with Z-VAD-FMK (20 mM; 4 h) followed by treatment with **2c** and analyzed for conversion of LC3A to LC3B, molecular marker for autophagy. Figure 13 suggests that in presence of apoptosis inhibitor, Z-VAD-FMK there was no significant changes observed in LC3B conversion in comparison to cells treated only with **2c**. Our result suggests that apoptosis and autophagy occur independently to each other in **2c** induced cytotoxicity.

## Discussion

Autophagy is a catabolic degradation process by its name (self eating) and mainly required to maintain cellular homeostasis. Autophagy constitutes multiple steps. It starts with formation of double membrane vesicles followed by engulfment of damaged proteins and organelles and then fusion of cargo-loaded autophagosomes with lysosomes which causes formation of autophagolysosomes in which the engulfed contents are broken down by acidic lysosomal hydrolases. Autophagy may play a dual role according to cellular environmental needs to contribute to cell survival and at the same time sometimes for cell death [41].

Nowadays increasing number of studies proposes autophagic cell death as a mechanism of action of some anticancer agents. These investigations suggest that autophagic cell death induction in cancers may have a therapeutic value. D Gozuacik et al. [42] remarked that molecules which are capable of inducing both autophagic cell death and apoptosis as ‘golden bullet’ since they are capable of triggering both caspase-dependent and autophagic, caspase-independent cell death at the same time. In this context, as we have previously reported that betulinic acid analogue, 2c is capable to induce mitochondria dependent pathway of apoptosis [43] and further we targeted to study another mode of cell death, autophagy which is one of the important constituent of cellular catabolic system. In addition, we focused on proteasomal degradation pathway also to investigate whole catabolic pathway after exploration of 2c on HT-29 cells. To the best of our knowledge, this is the first report which shows that a betulinic acid analogue has potential to induce autophagy in human colon carcinoma cells. In current cancer research the utmost requirement of successful cancer therapies demand development of drug without cytotoxicity to normal cells. Initially we screened 2c on various cancer cells and normal human PBMC. Analogue 2c was found most effective to selectively kill human colon carcinoma HT-29 cells with IC_50_ 14.9 μM and with negligible effect on normal human PBMC [43].

We directly perceived the percentage of autophagosome formation using Cyto-ID Green probes by flow cytometry and found an increasing percentage of autophagosome formation in 2c treated HT-29 cells in a time dependent manner (Fig. 1).

Earlier reports suggest that formation of autophagic vacuoles is an established marker of ‘autophagic cell death’ [44]. As we have shown in Fig. 2, Acrilidine Orange stains autophagic acidic vacuoles and emits red fluorescence after treatment with 2c for different time periods.

Moreover, we have validated progression of autophagy pathway by using Monodansylcadaverine (MDC), an autofluorescent compound which is reported to stain autophagic vacuoles. As shown in Fig. 3, analogue 2c treated HT-29 cells showed. a subtle increase in accumulation of MDC-labeled vacuoles.

We have endeavoured to measure different autophagy related protein induced by 2c in HT-29 cells like Beclin-1, a key regulatory protein of autophagy involved in nucleation step of autophagosome formation. Autophagy-related gene (Atg) products are another important regulator of autophagosome formation. Autophagy initiation is accompanied by amalgamation of Atg 12 to Atg 5 with the aid of Atg 7. We observed that 2c was capable to increase expression levels of Beclin-1, Atg 3, Atg 5, Atg 7, coalesced form Atg 5-Atg 12 and p62 (Fig. 4) as well as convertion of LC3A to LC3B (Fig. 5), the gold standard hallmark of autophagy [45] which were measure by western blot analysis. Cytosolic-associated protein light chain 3 (LC3A) remains conjugated with phosphatidylethanolamine [46]. In presence of Atg 3, LC3A is converted to the membrane bound lipidated form, LC3B.

To determine the contribution of 2c in induction of autophagic mode of cell death in HT-29 cells, 3-MA (interferes with autophagy initiation) was used. Interestingly, 3-MA was found to retrieve the viability of 2c treated HT-29 cells (Fig. 6). Autophagic flux can be monitored by LC3B turnover using western blot analysis in the presence and absence of lysosomal degradation inhibitors, such as pepstatin A, E64d, bafilomycin A1, chloroquine etc. [47]. It is reported that the level of LC3B is increased in the presence of the lysosomal degradation inhibitors because the degradation of LC3B through the autophagic pathway was hindered. In our study we demonstrated that LC3B level increased in the presence of each lysosomal degradation inhibitors in compare to control suggesting 2c is capable to induce autophagy in HT-29 cells (Fig. 7).

We have also shown time dependent up-regulation of Beclin-1 and LC3B mRNA expression level (Fig. 8).

Literatures suggest that detecting the presence of autophagic vesicles by using transmission electron microscopy (TEM) is the most sensitive and gold standard technique to monitor autophagy. Autophagosomes are typically identified as double membrane envelop vacuolar structures, sequestering cellular contents [48]. Our TEM data suggest that treatment with analogue 2c in HT-29 cells causes formation of double membrane autophagic vacuoles constitutively after 12, 24 and 48 h of treatment as compared to control HT-29 cells (Fig. 9). At 12 h of treatment few autophagosomes were seen but transient increase in the number of autophagosomes was noted after 24 and 48 h of 2c treatment in HT-29 cells. These membrane enclosed autophagic vacuoles become enlarged and eventually forms giant double membrane autophagosomes which finally fused with lysosomes resulting in the formation of autolysosomes. As we can see from the figure autophagosomes become enlarged at 24 h and at 48 h autolysosome formation was completed.

In our study we have also observed downregulation of caspase-like, trypsin like and chymotrypsin-like activities of the proteasomal enzyme after analogue 2c treatment (Fig. 10).

In support of the notion of altered expression of different autophagic proteins by western blotting, we also observed time dependent protein expression levels of Beclin-1, Atg 5, Atg 7, LC3B and p62 by immunoflourescence study using confocal microscopy (Fig. 11). Upon formation of LC3B, it continues to exist on mature autophagosomes till the complete association of autophagosomes with lysosomes. We aimed to investigate the colocalization of LC3B with lysosome. After 24 h treatment with 2c, cellular puncta which were positive for both red and green (merged image) were found in HT-29 cells (Fig. 12a).

Concurrently, p62 protein is considered as an adaptor molecule which contains dual binding site through which it can simultaneously interact with both LC3B as well as polyubiquitinated protein aggregates. p62 is usually degraded during autophagy as it is involved in trafficking of cellular ubiquitinated proteins to the proteasome or to autophagolysosome. We subsequently focused to investigate whether the adaptor molecule p62 is engaged in trafficking cellular ubiquitinated proteins to autophagolysosome.

In our study we observed that autophagy is crucially related with proteasomal degradation pathway which inspired us to find out 2c mediated interplay between autophagy and proteasomal degradation pathway through involvement of the ubiquitinated protein adapter molecule, p62. Emerging evidence indicates that proteasome dependent protein degradation was downregulated upon activation of autophagy which makes completion of polyubiquitinated proteins degradation via formation of autolysosome [49]. It is reported that the ubiquitin binding protein p62 which contain a ubiquitin-binding domain, can cargo the polyubiquitinated proteins to autolysosome [50]. In this milieu, we studied the co-localization of LAMP-1 with LC3B, LC3B with Lysosome, p62 with lysosome, ubiquitin with lysosome and LC3B with p62. We determined the percentage of cellular puncta co-localized for all five pairs of protein sets which indicated progression of autophagic pathway and clearance of cellular ubiquitinated proteins through formation of autophagolysosome (Fig. 12a, b).

In this study, our objective was to unveil the molecular mechanism towards betulinic acid analogue (2c) induced cell death in colon cancer cell. In view of the high potency of betulinic acid towards colon cancer, this compound has the potential to be exploited as a therapeutic agent in the adjunct therapy of colorectal cancer.

## Conclusions

On the basis of our present study we must state that our lead compound 2c, a potent betulinic acid analogue induces autophagy in human colon carcinoma, HT-29 cells at the same time averts proteasomal degradation pathway in parallel suggesting a novel crosstalk in between these two pathways. Recent studies have also shown that a regulation exists between the UPS and autophagy, the two cellular catabolic systems. We have already reported that 2c induces apoptosis in HT-29 cells in our previous study. So, all together our findings unveil that 2c is capable to induce both apoptotic as well as autophagic mode of cell death in HT-29 cells independently. Interestingly, as **2c** causes significant reduction in cell viability of both the colorectal adenocarcinoma cell lines (HT-29 and HCT-15), so it may prove itself to be a potential therapeutic agent for colon cancer, providing a basis for the development of the compound as a novel anticancer agent.

## Abbreviations

AO: acridine orange
BA: betulinic acid
CLSM: confocal laser scanning microscopy
MDC: monodansylcadaverin
PCD: programmed cell death
qRT-PCR: quantitative real-time PCR
TEM: transmission electron microscopy
UPS: ubiquitin proteasome system
